# Homotaurine Treatment Enhances CD4^+^ and CD8^+^ Regulatory T Cell Responses and Synergizes with Low-Dose Anti-CD3 to Enhance Diabetes Remission in Type 1 Diabetic Mice

**DOI:** 10.4049/immunohorizons.1900019

**Published:** 2019-10-21

**Authors:** Jide Tian, Hoa Dang, Karen Anne O’Laco, Min Song, Bryan-Clement Tiu, Spencer Gilles, Christina Zakarian, Daniel L. Kaufman

**Affiliations:** Department of Molecular and Medical Pharmacology, David Geffen School of Medicine at UCLA, University of California, Los Angeles, Los Angeles, CA 90095

## Abstract

Immune cells express γ-aminobutyric acid receptors (GABA-R), and GABA administration can inhibit effector T cell responses in models of autoimmune disease. The pharmacokinetic properties of GABA, however, may be suboptimal for clinical applications. The amino acid homotaurine is a type A GABA-R (GABA_A_-R) agonist with good pharmacokinetics and appears safe for human consumption. In this study, we show that homotaurine inhibits in vitro T cell proliferation to a similar degree as GABA but at lower concentrations. In vivo, oral homotaurine treatment had a modest ability to reverse hyperglycemia in newly hyperglycemic NOD mice but was ineffective after the onset of severe hyperglycemia. In severely diabetic NOD mice, the combination of homotaurine and low-dose anti-CD3 treatment significantly increased 1) disease remission, 2) the percentages of splenic CD4^+^ and CD8^+^ regulatory T cells compared with anti-CD3 alone, and 3) the frequencies of CD4^+^ and CD8^+^ regulatory T cells in the pancreatic lymph nodes compared with homotaurine monotherapy. Histological examination of their pancreata provided no evidence of the large-scale GABA_A_-R agonist–mediated replenishment of islet β-cells that has been reported by others. However, we did observe a few functional islets in mice that received combined therapy. Thus, GABA_A_-R activation enhanced CD4^+^ and CD8^+^ regulatory T cell responses following the depletion of effector T cells, which was associated with the preservation of some functional islets. Finally, we observed that homotaurine treatment enhanced β-cell replication and survival in a human islet xenograft model. Hence, GABA_A_-R agonists, such as homotaurine, are attractive candidates for testing in combination with other therapeutic agents in type 1 diabetes clinical trials.

## INTRODUCTION

Clinical trials of immunotherapies for type 1 diabetes (T1D) have shown insufficient efficacy such that it is now generally thought that combination treatments will be needed to achieve more effective T1D intervention (1, 2). γ-aminobutyric acid (GABA) is a nonprotein amino acid that is commonly synthesized by neurons in the CNS and used as a neurotransmitter. There are two types of GABA receptors (GABA-Rs) that are encoded by different gene families, and their activation induces different pathways; type A GABA-Rs (GABA_A_-Rs) are fast-acting chloride channels and type B GABA-Rs (GABA_B_-Rs) are slow-acting G-protein coupled receptors (3, 4). Like neurons, rodent and human T cells express GABA-Rs, specifically those of the GABA_A_-R family (5–10). We, and others, have shown that GABA can downregulate proinflammatory T cell responses while simultaneously promoting regulatory T cell (Treg) responses (5, 7, 9–16). GABA also downregulates inflammatory activities of APC (14, 17). We posited that immune cell GABA-Rs may serve the purpose of limiting inflammation in the CNS and that this mechanism could be taken advantage of pharmacologically to limit inflammation in the periphery (9). Indeed, we, and others, have shown that GABA administration could inhibit autoimmune disease in mouse models of T1D (5, 7, 9, 11–13) and rheumatoid arthritis (14) and reduce inflammation and disease severity in type 2 diabetes mouse models (15, 18, 19). The insulin-producing β-cell s of pancreatic islets also express GABA-Rs, both GABA_A_-Rs and GABA_B_-Rs (20–25). Administration of GABA, or GABA_A_-R– and GABA_B_-R–specific agonists, has been shown to promote β-cell survival and replication in diabetic mice and human islet xenografts (7, 11, 20, 22, 24–29). Collectively, these studies indicate that GABA-R activation has multiple desirable effects that could help prevent and treat T1D, thereby making these receptors promising drug targets.

Although GABA consumption appears to be safe (30–33), GABA’s pharmacokinetic properties may not be optimal for clinical use. In particular, GABA has a relatively short half-life in plasma [~20 min after i.v. or i.p. injection (32, 34–36)], and GABA’s affinity (EC_50_) for GABA_A_-Rs is relatively low [~50–400 μM (37, 38)], presumably so that it rapidly dissociates from its receptors. Accordingly, identifying other GABA_A_-R agonists that are safe and have good pharmacokinetics holds potential importance for clinical applications. Homotaurine (also known as 3-APS and tramiprosate) is a natural amino acid found in algae and may be a good candidate to fill that role. Homotaurine was identified as a compound that could interfere with the ability of soluble amyloid peptide to form fibrils in vitro, making it a therapeutic candidate for Alzheimer’s disease (39, 40). In preclinical studies, oral homotaurine limited amyloid plaque deposition in the brain of transgenic mice that overexpressed human amyloid protein (39,40). Preclinical and early clinical pharmacokinetic/pharmacodynamic and toxicity studies found that homotaurine was safe and able to cross the blood–brain barrier in mice and humans (40, 41). A large phase III clinical trial with 1052 individuals tested the ability of oral homotaurine to slow the progression of Alzheimer’s disease over 1.5 y. Although homotaurine treatment did not slow cognitive decline, it had an excellent safety profile and no treatment-related adverse CNS effects in this long-term study (42–44).

More recently, it has become appreciated that homotaurine is a GABA_A_-R agonist and has a higher affinity for GABA_A_-Rs than GABA as well as a longer half-life in plasma (≈3 h versus 20 min for GABA after i.v. or i.p. injection) (32, 34–36, 40). Homotaurine has little or no effect on GABA_B_-Rs; its IC_50_ for GABA_B_-Rs is 260-fold greater than that of the GABA_B_-R–specific agonist baclofen and is very similar in magnitude to that of the prototypic GABA_A_-R–selective agonist muscimol (45–47). We recently reported that oral homotaurine treatment inhibited murine experimental auto-immune encephalomyelitis (EAE), a model of multiple sclerosis (MS), and that this was accompanied by enhanced Treg responses (16). Whereas autoreactive Th17 responses are crucial for the pathogenesis of EAE and MS (48, 49), autoreactive Th1 responses are thought to play a major pathogenic role in murine and human T1D (50, 51). There is currently no information on whether homotaurine has the therapeutic potential to ameliorate Th1-mediated disorders such as T1D. Moreover, it is unknown whether a GABA_A_-R–specific agonist drug can induce beneficial effects in T1D as effectively as GABA (which activates both GABA_A_-R and GABA_B_-Rs). Finally, it is unknown whether this therapeutic strategy can be effective when given in combination with agents that deplete effector T cells, an interventive strategy that is being actively pursued in T1D clinical trials (52).

In this study, we assessed the therapeutic potential of homotaurine in NOD mice. Although many different treatments can prevent the onset of T1D in NOD mice, few have shown the capacity to reverse T1D after its onset in these mice. We observed that homotaurine monotherapy has a modest ability to reverse T1D in newly diabetic NOD mice. We then tested whether homotaurine treatment could augment the therapeutic efficacy of 1) an Ag-specific therapy (proinsulin/alum immunization) and 2) a therapy based on the depletion of effector T cells (low-dose anti-CD3) in newly diabetic and severely diabetic NOD mice, respectively. These treatment combinations were chosen for study based on their apparent safety and clinical potential. We show for the first time, to our knowledge, that a GABA-R agonist, homotaurine, can augment the effectiveness of a low-dose immunosuppressant (anti-CD3) and that this was accompanied by increased CD8^+^ and CD4^+^ Tregs in severely diabetic NOD mice. Finally, we show that treatment with homotaurine enhances the survival of human islet cells and promotes β-cell replication in human islet xenografts.

## MATERIALS AND METHODS

### Chemicals

Homotaurine (stock no. A76109), GABA, bicuculline, and BrdU were purchased from Sigma-Aldrich (St. Louis, MO).

### Mice

NOD and NOD/scid mice were originally from Taconic Biosciences (Germantown, NY) and maintained in our specific pathogen-free facility. Female NOD and NOD/scid mice were used in this study. This study was carried out in accordance with the recommendations of the Guide for the Care and Use of Laboratory Animals of the National Institutes of Health. The protocols for all experiments using vertebrate animals were approved by the Animal Research Committee at University of California, Los Angeles.

### T cell proliferation assays

Splenic mononuclear cells (3 × 10^5^/well) from naive mice were treated in triplicate with anti-CD3 (1 μg/ml, 2c11 clone; PharMingen, San Diego, CA) in the presence or absence of the indicated doses of GABA or homotaurine for 48 h. During the last 14 h of incubation, [^3^H]thymidine (1 μC/well) was added into each well to determine T cell proliferation. The data are presented as the mean [^3^H]thymidine incorporation ± SD of each group of cells relative to that in control cultures without homotaurine or GABA treatment.

### Homotaurine monotherapy in newly diabetic NOD mice

Beginning at 12 wk in age, female NOD mice blood glucose levels were monitored two to three times per week using a OneTouch ultrasensitive monitor. Animals with blood glucose levels between 250 and 300 mg/dL for two consecutive days were recruited into the study. The mice were randomized and treated with plain drinking water as the control or drinking water containing 0.08, 0.25, or 0.75 mg/ml of homotaurine (pH 7.2). Fresh homotaurine containing water was prepared each week. The animals’ blood glucose levels were monitored for up to 45–50 wk post-T1D onset. Individual mice with two consecutive blood glucose readings <250 mg/dL were considered to be in remission, after which two consecutive blood glucose readings >250 mg/dL was considered as disease relapse.

### Combined homotaurine and Ag-specific immunotherapy treatment

Newly diabetic mice (two consecutive blood glucose levels of 250–300 mg/dL) were treated with proinsulin (100 μg, kindly provided by Eli Lilly, Indianapolis, IN) in 50% alum (Pierce Biotechnology, Rockford, IL) i.p. The same day, the animals were placed on water containing homotaurine (0.25 mg/ml), which was continued throughout the experimental period. The mice were boosted with proinsulin/alum 10 d later and were monitored for disease remission and relapse as described above.

### Combined homotaurine and low-dose anti-CD3 treatment in severely diabetic NOD mice

To assess whether homotaurine could augment the efficacy of an effector T cell–depletive therapy after the establishment of severe hyperglycemia, we treated diabetic NOD mice that had a blood glucose reading of >340 mg/dL with low-dose anti-CD3 (35 μg hamster anti-CD3ε 2C11 F(ab′)_2_ fragment i.v., Bio X Cell, West Lebanon, NH) for three consecutive days, a treatment which was observed to be partially effective in our pilot studies (data not shown) and reports by von Herrath and colleagues (53). Mice with two consecutive blood glucose readings between 340 and 550 mg/dL within 1 wk of the initial treatment were included in our subsequent analysis. At the time of the first anti-CD3 treatment, the animals were randomized to receive plain water or water containing homotaurine (0.25 mg/ml, continuously) and monitored for disease remission and relapse for up to 25 wk. At 25 wk after initiating treatment, the pancreas from some surviving mice were processed for immunofluorescent staining with anti-insulin, antiglucagon, and DAPI, as previously described (13).

### i.p. glucose tolerance test

Twenty-five weeks after initiating treatment, some mice were fasted for 16 h, and their blood glucose was monitored just before as well as 15, 30, 60, 90, 120, and 180 min postchallenge with glucose (2 g/kg) i.p. The area under the curve of the blood glucose levels was calculated.

### Flow cytometry

At 25 wk posttreatment, some severely diabetic mice that responded to anti-CD3 monotherapy or combined anti-CD3^+^ homotaurine treatment were sacrificed, and their splenic mono-nuclear cells were isolated. Some splenic mononuclear cells were stained with FITC–anti-CD4, fixed, permeabilized, and then stained intracellularly with PE–anti-Foxp3. The percentages of splenic CD4^+^Foxp3^+^ Tregs in total CD4^+^ T cells were determined by flow cytometry. In addition, splenic mononuclear cells were stained with FITC–anti-CD8, APC–anti-CD122, and PE–anti–PD-1. The percentages of splenic CD8^+^CD122^+^PD-1^−^ and CD8^+^CD122^+^PD-1^+^ Tregs in total CD8^+^ T cells were determined by flow cytometry as previously described (16).

In a separate study, female NOD mice at 15–18 wk of age were randomized and injected i.v. with vehicle saline or a suboptimal dose of anti-CD3 for three consecutive days. The mice were also randomized and provided with water alone or water containing homotaurine (0.25 mg/ml) for 3 wk. Their pancreatic lymph nodes (PLNs) were isolated, and the mononuclear cells in the PLNs of each mouse were prepared. Subsequently, the percentages of CD4^+^Foxp3^+^ Tregs in total CD4^+^ T cells and CD8^+^CD122^+^PD-1^−^ and CD8^+^CD122^+^PD-1^+^ Tregs in total CD8^+^ T cells in the PLN of each mouse were determined by flow cytometry.

### Islet cell proliferation assay

Fresh human islets were obtained from the Integrated Islet Distribution Program. The islets (50–75 islet equivalents per well) were treated in triplicate with or without the indicated dosages of homotaurine in CMRL medium (0.1% glucose; Life Technologies, Grand Island, NY) containing 10% human AB-type sera (MP Biomedicals, Santa Ana, CA) and 1.5 μCi/ml of [^3^H]thymidine for 4 d. The [^3^H]thymidine uptake in individual wells was measured by beta counter. Data were analyzed by the proliferation index formula: cpm of experimental wells/cpm of controls.

### Analysis of human β-cell replication in vivo

All batches of human islets for transplantation and assessments of β-cell replication met the criteria of >90% viability, >85% purity, from a donor <45 y old, and were received within 36 h postisolation, which, in our experience, were optimal parameters for studying human β-cell replication in the xenograft model. Individual NOD/scid mice were injected i.p. with streptozotocin (STZ) to induce diabetes and implanted with ~2000 human islets under their kidney capsule. Two days later, the mice were randomized and treated for 10 d with plain water containing BrdU (0.8 mg/ml) as the control, water with homotaurine (0.08 or 0.25 mg/ml) and BrdU, or water containing GABA (6 mg/ml) and BrdU as a positive control. Their consumption of water and food was monitored. At the end of treatment, the percentages of BrdU^+^insulin^+^ and Ki67^+^insulin^+^ β-cell s in at least 2000 islet cells of 10 fields (magnification ×400) of each islet graft were determined by immunofluorescence in a blinded manner, as in our previous report (26).

### Analysis of human β-cell apoptosis in vivo

STZ-rendered diabetic NOD/scid mice were implanted with ~2000–3000 human islets under their kidney capsule. The mice were randomized and given plain water as the control, water containing homotaurine (0.08 or 0.25 mg/ml), or GABA (6 mg/ml) as the positive control. Forty-eight hours later, the percentages of insulin^+^ β-cell s or TUNEL^+^ apoptotic islet cells in total islet cells within the grafts of individual recipients were determined by immunofluorescence in a blinded manner, as in our previous report (26).

### Statistical analysis

Data are expressed as mean ± SD. The comparisons among different treatment groups were performed by the nonparametric Wilcoxon signed rank test or log-rank test. Other data were analyzed by one-way ANOVA, and post hoc least significant difference and the difference between two groups were analyzed by Student *t* test using the SAP software. A two-tailed *p* value <0.05 was considered statistically significant.

## RESULTS

### Homotaurine inhibits in vitro proliferative T cell responses to a similar degree as GABA

**FIGURE 1.**
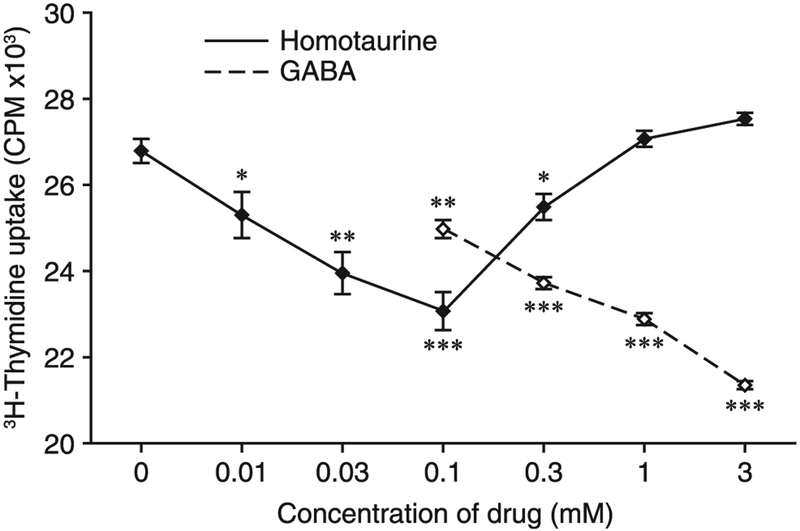
Homotaurine inhibits the proliferation of splenic T cells. Splenic mononuclear cells were stimulated in triplicate with anti-CD3 in the presence or absence of different doses of homotaurine or GABA for 48 h, and the proliferation of T cells was determined by [^3^H]thymidine uptake as described in *Materials and Methods*. Splenic mononuclear cells in medium alone had [^3^H]thymidine uptake at 600–850 cpm. *p < 0.05, **p < 0.01, ***p < 0.001 versus the anti-CD3–stimulated T cells without homotaurine or GABA, determined by Student *t* test.

We first assessed homotaurine’s ability to inhibit proliferative T cell responses in vitro. Splenic mononuclear cells were isolated from naive NOD mice and stimulated with anti-CD3 in the presence of different doses of homotaurine or GABA for 48 h. Treatment with GABA at 0.1–3 mM inhibited the proliferation of splenic T cells in a dose-dependent manner (Fig. 1). The effects of homotaurine treatment on splenic T cell proliferation displayed a “U-shaped” dose-response curve, with maximal inhibition occurring at 0.1 mM. The maximal inhibitory effect of homotaurine at 0.1 mM was of similar magnitude to that of GABA at 3–10-fold higher concentrations (0.3–1.0 mM).

### Homotaurine treatment reverses hyperglycemia in newly diabetic NOD mice

**FIGURE 2.**
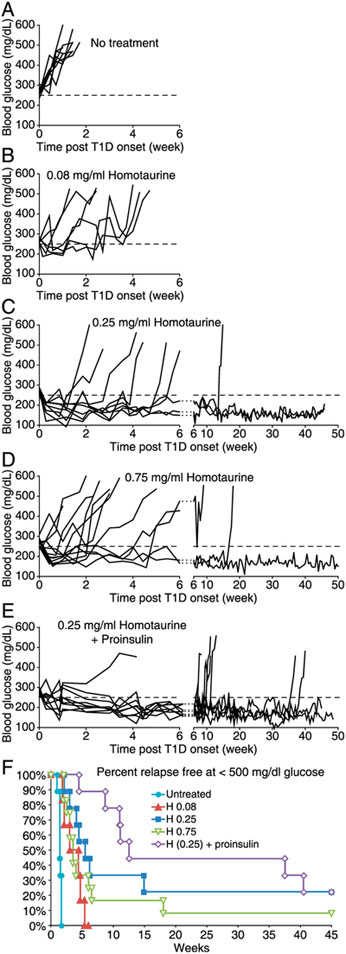
Homotaurine can reverse hyperglycemia in newly diabetic NOD mice. Newly diabetic NOD mice were (**A**) untreated or continually given homotaurine at (**B**) 0.08 mg/ml (*n* = 6), (**C**) 0.25 mg/ml (*n* = 9), or (**D**), 0.75 mg/ml (*n* = 12) through their drinking water. (**E**) In a subsequent study, newly diabetic NOD mice were treated with both homotaurine (0.25 mg/ml) and proinsulin/alum (*n* = 9). Data shown are longitudinal blood glucose levels for individual mice. Dashed line indicates blood glucose of 250 mg/dL. (**F**) Data shows percentage of relapse-free mice in each treatment group (blood glucose < 500 mg/dL). *p* ≤ 0.001 for all homotaurine treatments versus untreated mice. *p* = 0.02 for homotaurine at 0.25 versus 0.08 mg/ml, *p* = 0.12 for homotaurine at 0.25 versus 0.75 mg/ml, *p* = 0.19 for combined homotaurine (0.25) and proinsulin/alum treatments versus homotaurine monotherapy, determined by the log-rank test.

To determine whether homotaurine had the therapeutic potential to reverse hyperglycemia in NOD mice, newly diabetic NOD mice (blood glucose 250–350 mg/dL) were randomized and placed on plain water (controls) or water containing 0.08, 0.25, or 0.75 mg/ml of homotaurine. There was no significant difference in water or food consumption between these groups of mice (data not shown). The control mice that received plain water rapidly progressed to severe hyperglycemia within 1 wk (Fig. 2A). Most of the mice that received homotaurine at 0.08 mg/ml displayed a very brief remission period (mean of 2.2 wk, Fig. 2B). Treatment with homotaurine at 0.25 mg/ml rapidly restored normoglycemia in all mice (Fig. 2C). Most of these mice became hyperglycemic again within 6 wk posttreatment, but a few mice remained in remission for 14–46 wk (the end of the study), leading to a mean remission time period of 14 wk for all mice. Treatment with a higher dose of homotaurine (0.75 mg/ml) corrected hyperglycemia in most mice (Fig. 2D). The mice in remission generally redeveloped diabetes within 6 wk, but a few mice remained in remission for a longer period, leading to a mean remission period of 7.5 wk for all mice. Thus, oral homotaurine treatment at an appropriate dose can temporarily and, in a few cases, may permanently reverse hyperglycemia in newly diabetic NOD mice.

We next tested whether the combination of oral homotaurine (0.25 mg/ml) and proinsulin/alum immunization treatments could increase the frequency or length of disease remission in newly diabetic NOD mice. We have previously reported that proinsulin/alum immunization could not reverse disease in newly diabetic NOD mice (13). In this study, we observed that similar to homotaurine (0.25 mg/ml) monotherapy, all mice receiving the combined therapy displayed a period of disease remission (Fig. 2E). The mice receiving combined therapy had a mean remission period of 24 wk (Fig. 2E), which was an increase of 10 wk over the mean remission period following homotaurine monotherapy (14 wk, Fig. 2C), although this difference was not statistically significant. The percentage of relapse-free mice in all groups are shown in Fig. 2F.

### The combination of homotaurine and low-dose anti-CD3 treatment increases remission frequency in severely diabetic NOD mice

**FIGURE 3.**
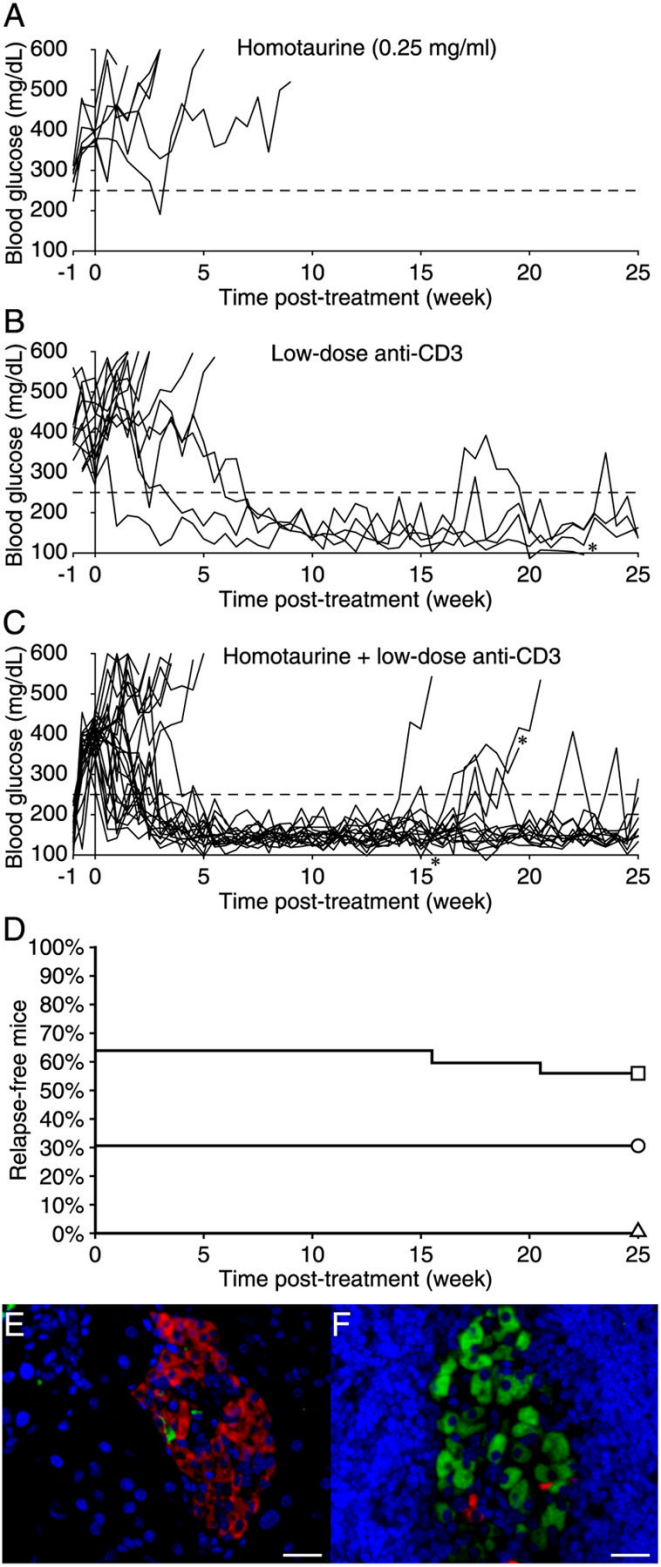
Combined treatment with homotaurine and low-dose anti-CD3 reverses hyperglycemia in severely diabetic NOD mice. Newly diabetic NOD mice with severe hyperglycemia were given (**A**) homotaurine (0.25 mg/ml, *n* = 7), (**B**) low-dose anti-CD3 (*n* = 13), or (**C**) combined low-dose anti-CD3 plus homotaurine (*n* = 25), as described in *Materials and Methods*. *Died of unknown causes. (**D**) Data shows percentage of relapse-free mice in homotaurine (triangle), anti-CD3 (circle), and anti-CD3 plus homotaurine treated mice (square) over the 25-wk observation period. Statistical analysis indicates homotaurine versus anti-CD3 plus homotaurine (*p* = 0.002) and anti-CD3 versus anti-CD3 plus homotaurine (*p* = 0.05) by the log-rank test. (**E**) A representative image of a nonfunctional islet observed in mice given anti-CD3 (alone) that was costained with anti-insulin (green), antiglucagon (red), and DAPI. (**F**) A representative image of a functional islet observed in mice given combined therapy. Scale bar, 25 μm.

Next, we tested the hypothesis that combining GABA-R activation with an immunosuppressant may allow effective disease reversal using lower dosages of the immunosuppressant, thereby reducing the possibility of its side effects. We chose to test anti-CD3 because it is a prototypic immunosuppressant that is in clinical use and has shown promise in T1D clinical trials (54–56). Because individuals are generally severely hyperglycemic at the time they are diagnosed with T1D, we focused on testing newly diabetic NOD mice with severe hyperglycemia. In pilot studies, we identified a low-dose anti-CD3 treatment protocol (three consecutive 35 μg treatments) that reversed hyperglycemia in about a third of the newly diabetic NOD mice. We then randomly treated newly diabetic NOD mice that had severe hyperglycemia with low-dose anti-CD3 and placed them on plain water or water containing homotaurine (0.25 mg/ml, the best dose from the monotherapy studies above). A control group received homotaurine alone. We observed that homotaurine monotherapy was unable to induce remission at this later stage of the disease(Fig. 3A). Low-dose anti-CD3 treatment led to disease remission in 31% of the treated animals, although in most cases, it took several weeks for remission to occur (Fig. 3B, 3D). Impressively, the combination of low-dose anti-CD3 and homotaurine doubled the remission rate (to 64%) compared with that of anti-CD3 treatment alone (*p* = 0.05 versus low-dose anti-CD3 monotherapy), and 82% of these mice remained in remission throughout the 25-wk observation period (Fig. 3C, 3D). Thus, the combination of low-dose anti-CD3 and homotaurine had an increased ability to restore and maintain normoglycemia after the development of severe hyperglycemia in NOD mice.

An i.p. glucose tolerance test was performed on the mice that remained in remission 25 wk after initiating treatment. We observed no significant difference between mice that received anti-CD3 alone and those that received combined therapy as might be expected because both these groups of mice were normoglycemic. Immunohistological analysis of pancreata from severely diabetic NOD mice that responded to low-dose anti-CD3 monotherapy revealed that their islets had just a few insulin^+^ cells when examined 25 wk after the initiation of treatment (a representative image is shown in Fig. 3E). These nonfunctional islets were essentially insulitis free. The vast majority of islets in pancreata from mice that had been started on combined therapy 25 wk earlier were also almost devoid of insulin^+^ cells, except that we observed rare islets that had many β-cell s (a representative image is shown in Fig. 3F). These functional islets had a surrounding peri-insulitis, and islet membrane damage was evident.

### Combination of homotaurine and anti-CD3 treatments increases the frequency of CD4^+^ and CD8^+^ Tregs in the spleens and PLNs of severely diabetic NOD mice

**FIGURE 4.**
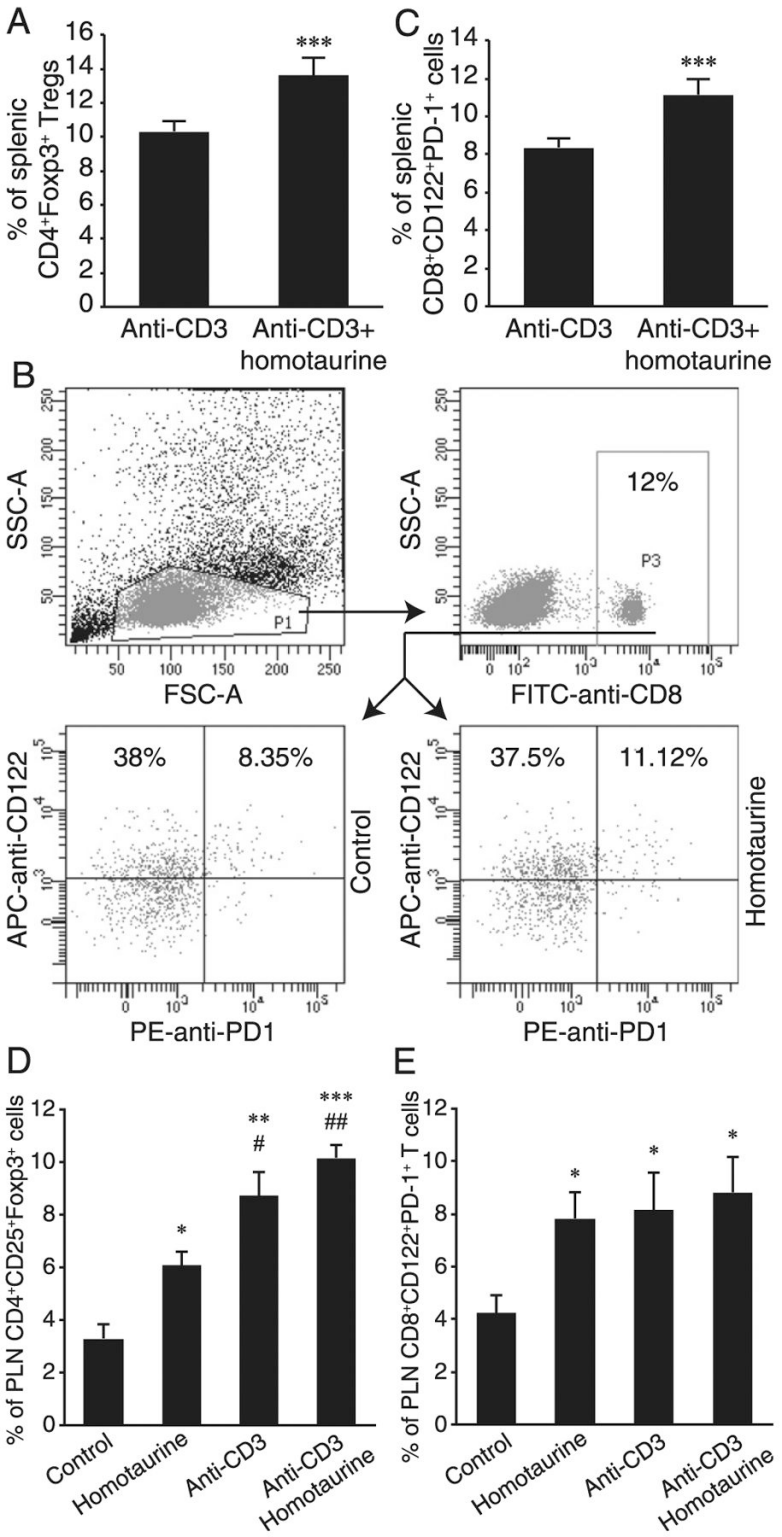
Combined treatment with homotaurine and low-dose anti-CD3 increases the frequency of splenic and PLN CD4^+^ and CD8^+^ Tregs. After treatment for 25 wk, the frequency of splenic CD4^+^FoxP3^+^ and CD8^+^CD122^+^PD-1^+^ Tregs in individual mice was determined by flow cytometry. The splenic mononuclear cells were gated first on living lymphocytes (data not shown), and the percentages of CD4^+^Foxp3^+^ Tregs were determined. In addition, the cells were gated on living lymphocytes and then on CD8^+^ T cells. The percentages of CD8α^+^CD122^+^PD-1^+^ Tregs were determined. Moreover, female NOD mice at 15–18 wk of age were randomized and treated i.v. with vehicle saline or a low dose of anti-CD3. The mice were randomized and provided with water (control) or water containing 0.25 mg/ml of homotaurine for 3 wk. Their PLNs were isolated, and the lymph node mononuclear cells were prepared. Subsequently, the frequency of PLN CD4^+^FoxP3^+^ and CD8^+^CD122^+^PD-1^+^ Tregs in individual mice was determined by flow cytometry. Data are representative flow cytometry charts of CD8^+^ Treg analysis and expressed as the mean ± SEM of each group (*n* = 4–5 mice per group) from two separate experiments. (**A**) Homotaurine increases splenic CD4^+^FoxP3^+^ Tregs in low-dose anti-CD3–treated mice. (**B** and **C**) Flow cytometry analysis of splenic CD8^+^CD122^+^PD-1^+^ Tregs. ***p < 0.001 versus the control with anti-CD3 alone. (**D** and **E**) Quantitative analysis of PLN CD4^+^FoxP3^+^ and CD8^+^CD122^+^PD-1^+^ Tregs. *p < 0.05, **p < 0.01, ***p < 0.001 versus the control with water. ^#^*p* < 0.05, ^##^*p* < 0.01 versus the mice receiving homotaurine alone.

Previous studies have shown that treatment with anti-CD3 depletes effector T cells while preserving splenic CD4^+^Foxp3^+^ Tregs in NOD mice (57). Our recent study with homotaurine in the EAE mouse model observed that homotaurine mono-therapy increased the frequency of splenic CD4^+^Foxp3^+^ and CD8^+^CD122^+^PD-1^+^ Tregs in SJL mice (16). It is unclear whether homotaurine has similar effects on Treg populations in the context of the Th1-biased genetic background of NOD mice and how combined treatment with anti-CD3 impacted Treg responses following the depletion of effector T cells in diabetic NOD mice. To begin to elucidate the potential mechanisms underlying the therapeutic action of combined homotaurine and anti-CD3, we first characterized the percentages of splenic CD4^+^Foxp3^+^ and CD8^+^CD122^+^PD-1^+^ Tregs in the mice 25 wk after treatment with anti-CD3 alone or combined anti-CD3^+^ homotaurine (Fig. 4A–C). We observed that the percentages of splenic CD4^+^Foxp3^+^ (Fig. 4A) and CD8^+^CD122^+^PD-1^+^ (Fig. 4B, 4C) Tregs in the mice given combined therapy were significantly higher than that of the mice given anti-CD3 monotherapy (*p* < 0.001 for both). There was no significant difference in the frequency of splenic CD8^+^CD122^+^PD-1^−^ T cells between these two groups of mice. Thus, combined treatment with homotaurine and anti-CD3 significantly increased the frequency of both CD4^+^ and CD8^+^ Tregs in the spleen in comparison with anti-CD3 monotherapy in severely diabetic NOD mice.

Analysis of PLN mononuclear cells in additional groups of NOD mice that had been treated with vehicle alone (control), homotaurine alone, low dose of anti-CD3 alone, or homotaurine^+^ anti-CD3 at 15–18 wk in age and studied 3 wk later showed that treatment with homotaurine or anti-CD3 monotherapies significantly increased the frequencies of CD4^+^CD25^+^Foxp3^+^ and CD8^+^CD122^+^PD-1^+^ cells in the PLN, relative to control groups (Fig. 4D, 4E). Notably, the combination treatment further elevated the average frequencies of CD4^+^CD25^+^Foxp3^+^ cells (Fig. 4D) and CD8^+^CD122^+^PD-1^+^ cells (Fig. 4E) in the PLN above that observed from the monotherapies (Fig. 4D, 4E). This increase was significant for CD4^+^CD25^+^Foxp3^+^ cells in comparison with homotaurine monotherapy. Thus, combined therapy also elevated the average frequencies of CD4^+^ and CD8^+^ Tregs within the target tissue/PLN of NOD mice.

### Homotaurine promotes islet cell replication in vitro through GABA_A_-Rs

**FIGURE 5.**
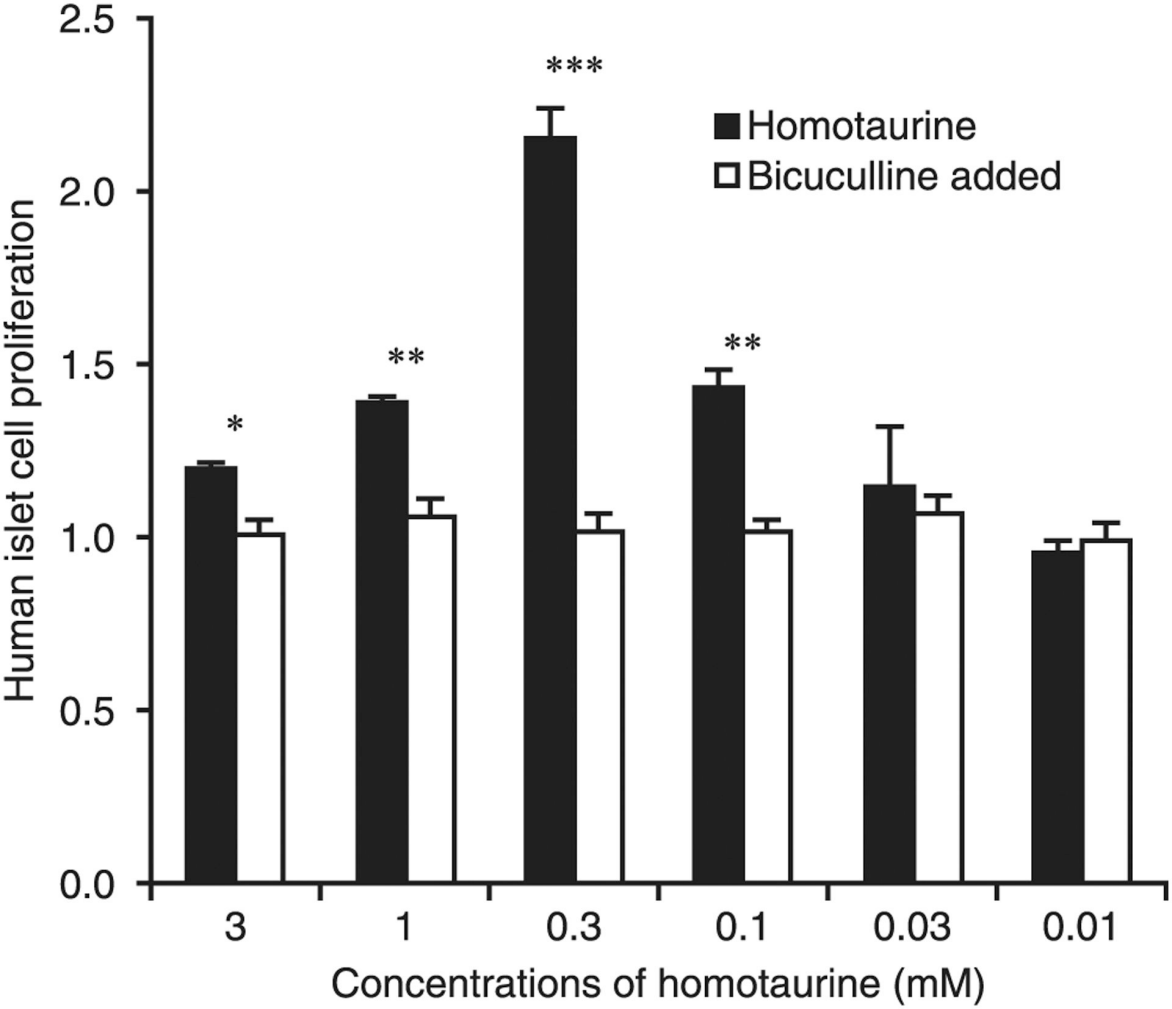
Homotaurine promotes human islet cell replication. Human islets were cultured in triplicate with or without the indicated dosages of homotaurine in the presence or absence of bicuculline for 4 d as described in *Materials and Methods*. Data shown are the mean ± SD proliferation index relative to that of cultures with medium alone (designated as 1) from two separate experiments. *p < 0.05, **p < 0.01, ***p < 0.001 for indicated homotaurine dose versus the control in medium alone, as determined by Student *t* test.

Cultured human islets were treated with or without different concentrations of homotaurine for 4 d, and the proliferation of islet cells was determined by [^3^H]thymidine incorporation. Treatment with 0.1–3 mM homotaurine significantly increased the proliferation index of the human islet cells (Fig. 5). The dose-response curve followed an inverted U-shape, with 0.3 mM homotaurine resulting in the highest proliferation index. The addition of bicuculline (50 μM, a GABA_A_-R antagonist) to the human islet cultures almost completely inhibited homotaurine’s ability to stimulate proliferation across the tested homotaurine dose range (Fig. 5), indicating that homotaurine-stimulated islet cell proliferation was highly dependent on GABA_A_-R activation. Hence, homotaurine-mediated activation of GABA_A_-Rs promotes human islet cell proliferation in vitro.

### Homotaurine promotes β-cell replication in human islet xenografts

To assess the nature of the replicating islet cells, we next used a human islet xenograft model and immunofluorescently examined β-cell replication at the single-cell level. NOD/scid mice were rendered diabetic with STZ and implanted with ~2000 human islets under their kidney capsule. Within 2 d, all islet graft recipients became normoglycemic and maintained normoglycemia throughout the experimental period. Two days after transplantation, the graft recipients were randomly placed on water containing BrdU alone (controls) or water containing homotaurine (at 0.08 or 0.25 mg/ml) or GABA (6 mg/ml, positive control) along with BrdU for 10 d. There was no significant difference in food and water consumption or body weights between these groups of mice (data not shown).

**FIGURE 6.**
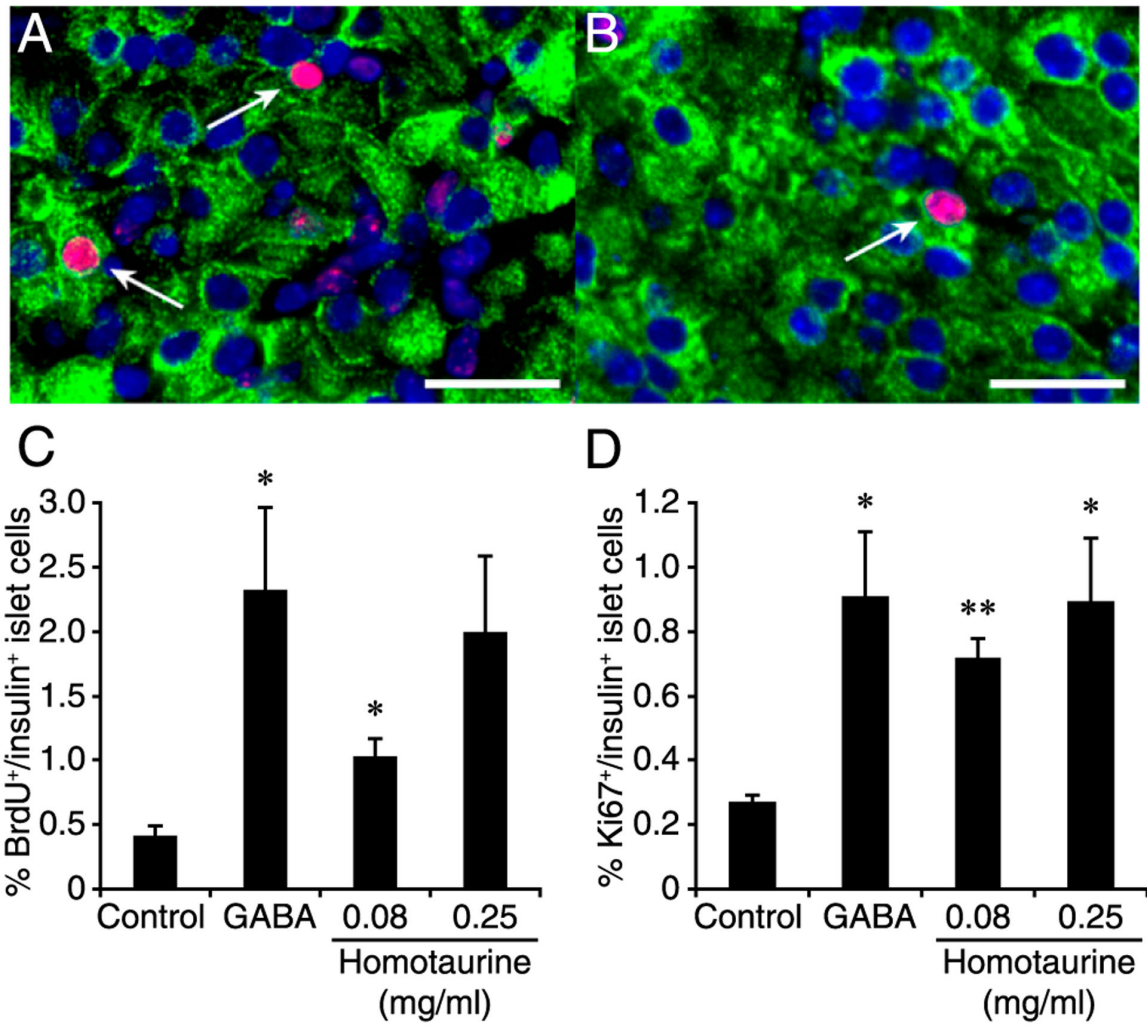
Oral homotaurine enhances human islet β-cell replication in a xenograft model. Hyperglycemic NOD/scid mice were transplanted with human islets under their kidney capsule. Within 2 d, all islet recipients had become normoglycemic and were then randomized to receive water containing BrdU, with or without homotaurine (0.08 or 0.25 mg/ml) or GABA (6 mg/ml) for 10 d. The percentages of replicated β-cell s were determined by immunofluorescent assays using Alexa Fluor 488–conjugated anti-insulin and Alexa Fluor 590–conjugated anti-BrdU or anti-Ki67, followed by counterstaining with DAPI. Representative images of islet cells (original magnification ×400) costained with (**A**) anti-insulin (green) and anti-BrdU (red, arrows) or (**B**) anti-insulin and anti-Ki67 (red, arrows). Scale bar, 25 μm. (**C**) Quantitative analysis of the percentages of insulin^+^BrdU^+^ cells in human islet xenografts in mice given plain water, GABA, or homotaurine. *p* = 0.07 and 0.7 for GABA versus homotaurine at 0.08 or 0.25 mg/ml, respectively. (**D**) Quantitative analysis of the percentages of insulin^+^Ki67^+^ cells in human islet xenografts. Data are mean ± SD from two independent studies with *n* = 4–9 implants analyzed per group. *p < 0.05, **p < 0.01 versus the control, determined by one-way ANOVA and post hoc least significant difference and Student *t* test.

Immunofluorescent analysis of the grafted islets revealed that oral homotaurine treatment at both 0.08 and 0.25 mg/ml significantly increased the frequency of BrdU^+^insulin^+^ and Ki67^+^insulin^+^ islet β-cell s relative to that in the islet grafts from the control mice that received plain water (Fig. 6). There was no significant difference in the frequency of BrdU^+^insulin^+^ or Ki67^+^insulin^+^ islet β-cell s among groups of mice treated with GABA or either dose of homotaurine (Fig. 6). Thus, homotaurine treatment increased human islet β-cell replication in vivo at levels similar to that of GABA.

### Homotaurine treatment improves human islet cell survival following islet transplantation

**FIGURE 7.**
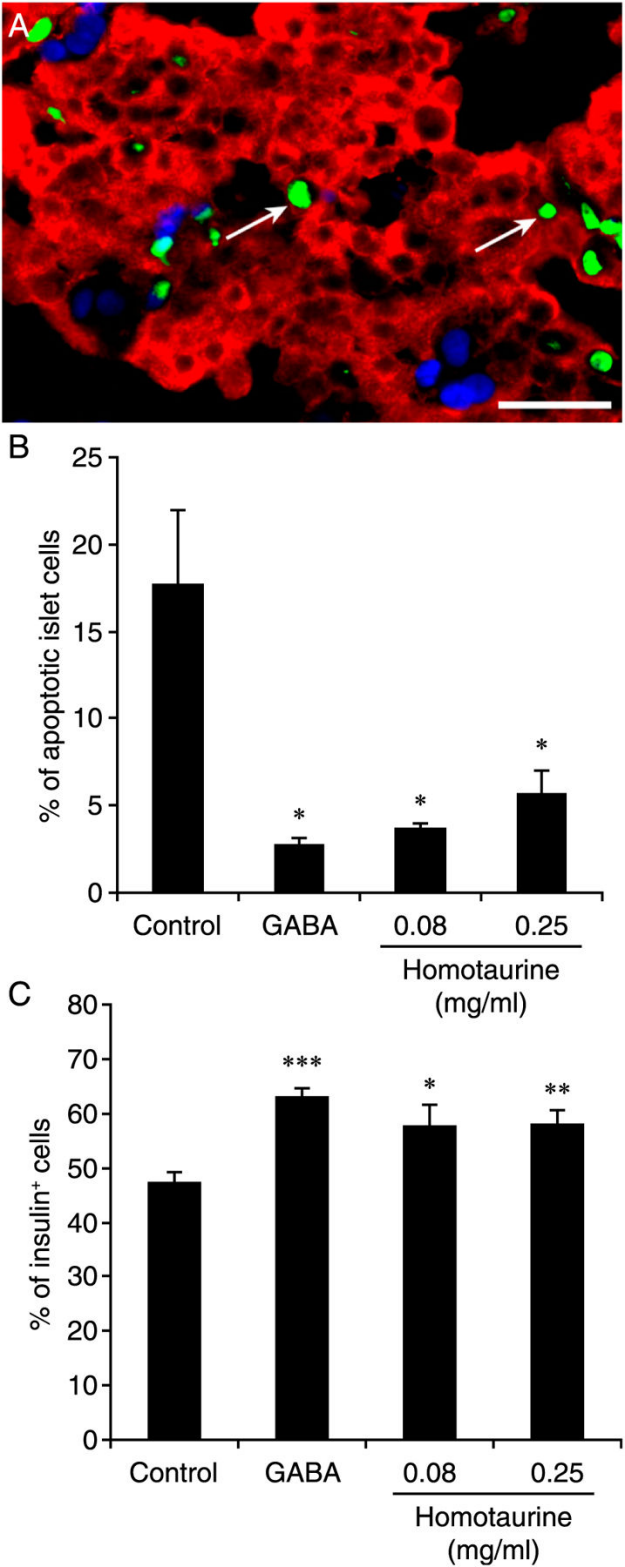
Homotaurine treatment protects human islet β-cell s from apoptosis in islet xenografts. Diabetic NOD/scid mice were implanted with human islets and, the next day, were placed on plain water, water containing homotaurine (0.08 or 0.25 mg/ml), or water containing GABA (6 mg/ml) for 48 h. The percentages of apoptotic human islet cells and remaining islet β-cell s in total human islet cells were determined by FITC-based TUNEL assay and costaining with Alexa Fluor 590–conjugated anti-insulin and DAPI. At least 2000 human islet cells in 10 fields (original magnification ×400) from individual grafts were counted. Data are representative images or expressed as the mean percentage ± SEM for each group of mice (*n* = 5–7) from three separate experiments. (**A**) A representative image with white arrows indicating TUNEL^+^ cells (green for TUNEL^+^, red for anti-insulin^+^, light blue for DAPI staining). Scale bar, 25 μm. (**B**) Quantitative analysis of the percentages of apoptotic islet cells and (**C**) insulin^+^ β-cell s. The difference among groups was analyzed by ANOVA and post hoc least significant difference and Student *t* test. *p < 0.05, **p < 0.01, ***p < 0.001 versus the control.

The process of human islet isolation and implantation cause a wide range of stressors that lead to the apoptosis of grafted islet cells within a few days following implantation (58, 59). We examined whether homotaurine administration could limit islet cell apoptosis in human islet xenografts. STZ-rendered hyperglycemic NOD/scid mice were implanted with human islets under their kidney capsule and received plain water (controls) or water containing homotaurine (0.08 or 0.25 mg/ml) or GABA (6 mg/ml, positive control) for 2 d. The implanted kidneys were then removed, and the graft tissue sections were subjected to TUNEL analysis, followed by anti-insulin staining (Fig. 7A). Treatment with 0.08 or 0.25 mg/ml of homotaurine significantly reduced the percentages of apoptotic islet cells (Fig. 7B) and increased the frequency of insulin^+^ β-cell s in the human islet grafts (Fig. 7C). On average, GABA-treated animals had less frequent apoptotic islet cells and a higher percentage of insulin^+^ cells than did homotaurine recipients, but these differences were not statistically significant. Such data indicated that homotaurine treatment improved human islet cell survival in a xenograft model.

## DISCUSSION

Efforts to develop cures for T1D aspire to safely reestablish β-cell tolerance and normoglycemia. Toward this goal, we conducted studies with homotaurine, a GABA_A_-R–specific agonist that appears to have an excellent safety profile and whose pharmacodynamics properties could have advantages over GABA for clinical use. To assess the therapeutic potential of homotaurine, we first tested its ability to inhibit proliferative anti-CD3–stimulated T cell proliferation in vitro. We observed that homotaurine inhibited mitogenesis in cultured splenic T cells at lower concentrations than GABA, but its inhibitory action followed a U-shape dose-response curve, whereas GABA displayed a linear dose-dependent response. Such U-shape dose-response curves are commonly observed in pharmacological investigations (60). T cells have relatively high intracellular Cl^−^ levels, and the opening of GABA_A_-R channels results in Cl^−^ efflux, membrane depolarization, and inhibition of Ca^2+^ entry (7, 9). Ca^2+^ influx is required for the activation of naive T cells and effector T cell function (61). Consistent with the notion that the activation of lymphocytic GABA_A_-Rs inhibits the T cell activation cascade, GABA-R activation arrests T cell cycling predominantly in the G_0_/G_1_ phase (9). This inhibition of T cell cycling is likely to contribute to homotaurine’s ability to quickly control inflammatory T cell responses in vivo, as further discussed below.

We then treated newly diabetic NOD mice with different doses of homotaurine. At the best dose tested (0.25 mg/ml), oral homotaurine reversed hyperglycemia for a median time period of 14 wk with a few of the mice remaining normoglycemic for the 46-wk observation period. These dosing studies suggest that homotaurine may have a relatively narrow effective dose range such that careful dosing studies will be necessary if clinical trials with homotaurine are pursued. It is possible that part of homotaurine’s antidiabetic effect could be through 1) its effects on CNS neurons or 2) the modulation of islet hormone secretion through GABA_A_-Rs on β-cell s or α-cells (20–25). We believe, however, that homotaurine’s major antidiabetogenic action is through quickly inhibiting the autoimmune responses that would otherwise rapidly destroy the residual β-cell s in newly diabetic NOD mice. Support for this contention includes the following: 1) treatment with GABA, which does not pass through the blood–brain barrier, can effectively inhibit the rapid destruction of β-cell s following the adoptive transfer of highly pathogenic mononuclear cells from newly diabetic NOD mice to NOD.scid recipients (9); and 2) oral homotaurine was moderately effective in inducing disease remission in moderately hyperglycemic mice but not severely hyperglycemic NOD mice, suggesting that α-cell hormones were not major mediators of disease remission. It is also worth noting that GABA-R agonists are effective immune modulators and therapeutics in models of rheumatoid arthritis and MS (14, 16), whose pathogenesis are distinct from that of T1D.

Combined treatment with homotaurine and proinsulin/alum treatments increased the mean time of disease remission from 14 wk (for the best dose of homotaurine monotherapy) to 24 wk, although this did not reach statistical significance. About half of the mice receiving combined therapy relapsed before 12 wk posttreatment. Because it takes some time for proinsulin/alum immunization to induce Treg immune responses and NOD mice progress rapidly from mild to severe hyperglycemia, it may be inherently difficult for autoantigen immunization to build up regulatory immune responses to preserve residual β-cell mass in this model. This may account for the inability of autoantigen immunization (alone) to reverse disease in newly diabetic NOD mice and their ability to augment the therapeutic efficacy of fast-acting immuno-modulators [e.g., homotaurine (shown in this study) and GABA (13)]. Our observations support the notion put forth by many in the T1D field that interventions should be initiated at presymptomatic stages of T1D (62). It will be of interest to determine whether earlier treatment, or additional boosting with proinsulin/alum after disease onset, may increase the therapeutic effect of combined therapy in our model.

Because human patients are generally severely hyperglycemic at the time of T1D diagnosis, we next tested the therapeutic effect of combined homotaurine and suboptimal doses of anti-CD3 in severely diabetic NOD mice. We observed that at this advanced stage of the disease, homotaurine monotherapy had little ability to restore normoglycemia. Low-dose anti-CD3 treatment led to disease remission in 31% of the treated animals, although this generally took several weeks to occur (Fig. 3B). In contrast, the combination of low-dose anti-CD3 and homotaurine more than doubled the remission rate (to 64%) compared with anti-CD3 treatment alone, with 82% of the responding mice remaining normoglycemic throughout the observation period. The rapid increase in T1D remission occurring shortly after administering combined therapy may reflect homotaurine’s ability to rapidly inhibit effector T cells, modulate APCs in PLN that will render them more tolerogenic, and/or its antiapoptotic properties that can act to preserve residual β-cell s. This is the first demonstration, to our knowledge, that the therapeutic efficacy of an effector T cell–depletive regimen can be enhanced by concurrent GABA-R activation and that such combinations may allow lower dosages of the effector T cell–depletive agent to be effective, thereby reducing its potential side effects.

Analysis of pancreata from severely diabetic mice that responded to low-dose anti-CD3 monotherapy and harvested 25 wk after the initial treatment revealed that the examined islets had few, if any, insulin^+^ cells. It is curious how these animals remained normoglycemic with so few remaining β-cell s; however, similar observations have been made in other studies of interventive therapies that were initiated after the onset of severe hyperglycemia in NOD mice [e.g., (63)]. The vast majority of islets in the mice given combined anti-CD3 and homotaurine treatments also had few, if any, insulin^+^ cells, with the exception that we observed rare islets that had many β-cell s. These functional islets had a surrounding peri-insulitis, a feature of functional islets that has been observed in past studies of anti-CD3–treated diabetic mice (53, 64, 65). We believe that these few functional islets reflect the ability of homotaurine to rapidly inhibit effector T cell responses and inhibit β-cell apoptosis, thereby helping to preserve the few remaining functional islets at the time when combined treatment was initiated. This contention, however, requires further studies with transgenic mice with which β- and α-cells can be genetically marked to identify pre-existing β- and α-cells and those arising after therapy because of replication, transdifferentiation, or neogenesis. The marking of pre-existing β-cell s will also address the possibility that GABA-R activation can promote the functional recovery of degranulated β-cell s, which could appear to be an increase in β-cell mass. Although the model we used may not be capable of excluding a low level of β-cell replenishment through transdifferentiation and neogenesis, the very high frequency of islets with few, if any, β-cells that we observed in mice given combined homotaurine and anti-CD3 treatment argues against the notion that GABA_A_-R agonists can induce the large-scale replenishment of β-cell s in diabetic mice as suggested by others (66, 67).

The few residual β-cell s in the islets of mice that had been severely diabetic make this model ill-suited for studies of GABA-R agonist’s ability to induce β-cell replication because these agonists increase the frequency of replicative β-cell s by only about 2–3-fold over the baseline level of <1% in adult mice [reviewed in (68)]. However, we, and other groups, have observed GABA-enhanced β-cell replication in newly diabetic mice, which generally have a larger residual β-cell mass (11, 26, 69). In this study, we observed that homotaurine enhanced the replication of β-cells severalfold in human islet xenografts, similar to past observations of GABA’s actions on human islet xenografts (26, 27). Notably, long-term studies of a minimal mass of human islets implanted into immune-deficient diabetic mice showed that GABA’s ability to enhance β-cell replication did not attenuate over time and led to an eventual increase in β-cell mass (27).

The question arises as to why commonly prescribed benzodiazepines such as Xanax (alprazolam) are not known to have antidiabetic effects. Actually, alprazolam was found to lower HbA1C in a small clinical trial with individuals with T1D and type 2 diabetes (70). Second, alprazolam is a positive allosteric modulator that prolongs the opening of the GABA_A_-R chloride channel only after GABA-mediated activation. Because of β-cell loss, there may be insufficient GABA in the islets of those with T1D to effectively modulate residual β-cell GABA-Rs. Notably, we observed that alprazolam treatment significantly reduced islet cell apoptosis and increased β-cell replication within human islet xenografts (71). Evidently, this GABA_A_-R positive allosteric modulator works in conjunction with GABA secreted from functional islet β-cell s to increase β-cell survival and replication. Combined treatment with alprazolam and GABA further enhanced human β-cell replication in the xenografts. Alprazolam also augmented the ability of suboptimal doses of GABA to inhibit Ag-specific T cell responses in vitro (71).

Our previous study with homotaurine in the EAE mouse model of MS observed that homotaurine inhibited the development of splenic autoantigen-specific Th1 and Th17 cells and increased the frequency of CD4^+^Foxp3^+^ and CD8^+^CD122^+^PD-1^+^ Tregs (16). Previous studies of anti-CD3 monotherapy observed that this treatment depleted CD4^+^Foxp3^−^ T cells while preserving, but not increasing, CD4^+^Foxp3^+^ Treg responses (57). Interestingly, we observed that the combination of low-dose anti-CD3 and homotaurine significantly increased the frequencies of splenic and PLN CD4^+^Foxp3^+^ and CD8^+^CD122^+^PD-1^+^ Tregs in NOD mice, extending our previous observations in the EAE mouse model (16). Currently, there is little information on how to induce CD8^+^ Tregs. Our results in this study, along with those of our previous EAE studies (16), suggest that GABA_A_-R agonists may provide a new treatment strategy to induce CD8^+^ Tregs in conditions involving deleterious inflammation.

The ability of homotaurine to inhibit inflammatory Th1 and Th17 responses while simultaneously enhancing CD4^+^ and CD8^+^ Tregs may be due to the different membrane potentials of these different T cell populations. Th17, Th1, Th2, and Tregs express distinct calcium channel subtypes and have different Ca^2+^ signaling patterns, with Th1 and Th17 cells showing the greatest Ca^2+^ influx, increased NFAT phosphorylation, and nuclear localization as well as cell motility, whereas Tregs display the least of these responses (72–74).

Our studies of homotaurine’s effects on cultured human islets showed that homotaurine increased islet cell proliferation with an inverted U-shaped dose-response curve. This promitotic effect was abrogated by the GABA_A_-R antagonist bicuculline, consistent with the notion that homotaurine promoted human islet cell proliferation through GABA_A_-R activation. Homotaurine also increased β-cell replication within human islet xenografts as measured by both BrdU/insulin and Ki67/insulin immunostaining. We focused on these readouts because the low level of homotaurine-induced increase in β-cell replication (~2% over controls as measured by BrdU/insulin^+^ staining) would be difficult to detect as a change in β-cell mass within the 10-d study time frame. The mechanisms by which GABA_A_-R activation promotes β-cell replication and survival are thought to be mediated by the opening of their Cl^−^ channels, which causes membrane depolarization and Ca^2+^ influx. This leads to activation of the PI3K/Akt pathway and the enhancement of β-cell replication and survival (11, 22, 27, 75, 76). The increased β-cell s are unlikely to arise from the transdifferentiation of human islet α-cells into β-cell s because 1) that was observed only after long-term GABA treatment (2–3 mo, as opposed to the 10-d treatment period in our xenograft studies) (66, 67), and 2) those observations have been called into question by others (77, 78) as well as by our observations in mice given combination therapy (discussed above).

We also observed that homotaurine treatment significantly preserved human islet cells from apoptosis in human islet grafts. Because a large percentage of β-cell s die shortly after transplantation because of stress-related apoptosis, short-term homotaurine treatment may be a promising adjunct therapy to help preserve β-cell mass following islet grafting and to reduce the number of islets necessary to achieve insulin independence.

In summary, our studies provide preclinical evidence that the GABA_A_-R–specific agonist homotaurine has a modest ability to reverse hyperglycemia in newly diabetic NOD mice. The combination of homotaurine and suboptimal doses of an immunosuppressive agent greatly increased the rate of remission in severely diabetic NOD mice, and this was accompanied by increased CD4^+^ and CD8^+^ Tregs in the periphery and target tissues. In addition, homotaurine treatment increased human β-cell survival and replication in vivo. Given that homotaurine has been shown to be safe in long-term phase III Alzheimer’s disease clinical studies, homotaurine treatment appears to be a promising adjunctive therapy for T1D intervention.

## Abbreviations used in this article:

EAE: experimental autoimmune encephalomyelitis
GABA: γ-aminobutyric acid
GABA_A_-R: type A GABA-R
GABA_B_-R: type B GABA-R
GABA-R: GABA receptor
MS: multiple sclerosis
PLN: pancreatic lymph node
STZ: streptozotocin
T1D: type 1 diabetes
Treg: regulatory T cell
